# Data-Weighted Multivariate Generalized Gaussian Mixture Model: Application to Point Cloud Robust Registration

**DOI:** 10.3390/jimaging9090179

**Published:** 2023-08-31

**Authors:** Bingwei Ge, Fatma Najar, Nizar Bouguila

**Affiliations:** Concordia Institute for Information Systems Engineering, Concordia University, 1515 St. Catherine Street West, Montreal, QC H3G 2W1, Canada; bi_ge@encs.concordia.ca (B.G.); f_najar@encs.concordia.ca (F.N.)

**Keywords:** multivariate generalized Gaussian, weighted-data clustering, minimum message length, point set robust registration, KL divergence, stochastic optimization

## Abstract

In this paper, a weighted multivariate generalized Gaussian mixture model combined with stochastic optimization is proposed for point cloud registration. The mixture model parameters of the target scene and the scene to be registered are updated iteratively by the fixed point method under the framework of the EM algorithm, and the number of components is determined based on the minimum message length criterion (MML). The KL divergence between these two mixture models is utilized as the loss function for stochastic optimization to find the optimal parameters of the transformation model. The self-built point clouds are used to evaluate the performance of the proposed algorithm on rigid registration. Experiments demonstrate that the algorithm dramatically reduces the impact of noise and outliers and effectively extracts the key features of the data-intensive regions.

## 1. Introduction

The purpose of point cloud registration is to extract the key points or features corresponding to the target point set and the point set to be registered as well as find the transformation mapping relationship between two point sets [1,2,3,4,5]. This task involving image processing, data analysis and computer vision has essential applications in many practical scenarios.

For instance, point cloud registration is essential for driverless technology. Indeed, various hardware sensors, such as lidars, short-wave radars and depth-of-field cameras, could be mounted on the crew-less vehicle and point cloud registration technology can be used to fuse data collected from multiple sensors [6,7,8] to provide fundamental functions such as scene stitching, vehicle positioning [9], and typical scene recognition and matching for vehicle control strategies. For example, the authors in [10] proposed a framework used for unmanned vehicles based on end-to-end point cloud registration deep networks. They obtained the corresponding relationship by learned matching probabilities (LMP) among a group of candidate points related to static characteristics instead of using existing points. Point cloud registration is also applied in medical imaging [11,12]. Indeed, to facilitate the diagnosis of the disease, several medical images from different instruments, such as Positron Emission Tomography (PET), Computed Tomography (CT) and Magnetic Resonance Imaging (MRI), need to be combined [13]. For example, authors in [14] improved the popular iterative Closest Point (ICP) algorithm by combining 3D scale-invariant feature transform to register 3D free-form closed surfaces (human skull models). In another work, the authors in [15] used the Gaussian mixture model (GMM) with a semi-supervised EM algorithm and geometric constraints to achieve retinal image registration. Moreover, 3D reconstruction also makes extensive use of point cloud registration technology [16,17]. For large buildings, for example, general scanning equipment cannot complete the whole scanning process at one time because the scanning range is limited. It requires scanning multiple parts and then splicing point clouds together [18]. In other cases, the objects to be observed may be dynamic or have complex surface characteristics. The accuracy of these features plays a crucial role in modelling and analysis; repeated scanning to build a fusion model can improve details [19].

Considering the point set’s acquisition perspective, noise and outliers generated in the acquisition process, as well as the deformation and missing parts of the point set caused by other factors, point cloud registration is a challenging task [1]. Various methods are proposed to enhance the robustness and accuracy of point cloud registration. In terms of pairwise registration, considering only two point sets, there are three main registration categories: distance-based methods including ICP [20], Graph Matching (GM) [21], filter-based methods and probability-based methods [22].

However, most point-to-point methods are prone to fall into local optima, especially if there are some similar point structure blocks in the point set. To improve this situation, registration based on mixed models (most are GMM-based) has proven effective [22,23,24,25]. The core idea is to model and describe the probability distribution of the point set using a parameterized mixed model and find the closest response of the mixture model to determine the corresponding relationship between point sets. These models perform well even if the sampling rate of two point clouds is not the same.

Nonetheless, there are two evident deficiencies when using the GMM. First, the GMM cannot effectively describe certain non-Gaussian distributions, such as the typical peak-trailing distributions in signal processing [26,27,28,29]. In the point cloud, intuitively speaking, space with dense data will carry more information. These high-density areas may represent the crucial feature structures in the point cloud, yet the GMM cannot effectively fit these high-density blocks, and its results tend to be relatively average. Secondly, GMM is easy to be disturbed by noise. Different noise levels will result in divergent response parameters in the mixture model, which could compromise the final registration accuracy [30].

The goal of this paper is to propose a point cloud registration method based on the weighted multivariate generalized Gaussian mixture model (WMGGMM) that we develop in this paper to address the difficulties above. The generalized Gaussian distribution (GGD) belongs to the family of elliptic distributions. Due to the addition of a shape parameter, it has a strong ability to describe various data distributions, particularly considering the data peak [31,32]. Its special cases contain Gaussian and Laplace distributions, and therefore it is widely used in feature extraction [33,34,35] and texture retrieval [36,37,38]. The mixture model of GGD has also been applied in several applications such as image processing and segmentation [39,40,41] and human movement recognition [42,43].

We show that the generalized Gaussian mixture model (GGMM) is an alternative worthy choice for point cloud registration when real-time analysis is not required. Although most parameters of GGMM have no closed solutions, its offers high registration accuracy and robustness. In addition, we introduce weights to reinforce the ability to pay attention to dense areas and reduce the influence of noise and outliers on the parameter estimation process. After obtaining the GGMM models for the target scene and the scene to be registered, the approximate Kullback–Leibler divergence (KLD) is computed to measure the models’ difference. This will be used as a loss function to find the optimal registration parameters through the stochastic optimization algorithm.

The paper is organized as follows. Section 2 will tackle the WMGGMM parameter estimation. This section will also give the complete learning algorithm. Section 3 presents some experimental results conducted on a synthetic data set to verify the algorithm’s performance and robustness. It is also devoted to our approach to obtain the optimal registration parameters using stochastic optimization, and the final registration effect performed on rigid transformation is presented. Conclusions and future works are finally reported in Section 4.

## 2. Weighted Multivariate Generalized Gaussian Mixture Model

In the majority of existing works, the feature independency assumption is used to simplify modeling high-dimensional data, which results in a distribution which is a product of one-dimensional generalized Gaussians [41,42]. Unlike these works, we use here the PDF as defined in [31]:

(1) $$p\left(x;\mu ,\Sigma ,\beta ,m\right)=\frac{\Gamma \left(\frac{d}{2}\right)\beta }{\Gamma \left(\frac{d}{2\beta }\right)\pi^{\frac{d}{2}}2^{\frac{d}{2\beta }}m^{\frac{d}{2}}{\left|\Sigma \right|}^{\frac{1}{2}}}e^{-\frac{1}{2m^{\beta }}{\left({\left(x-\mu \right)}^{T}\Sigma^{-1}\left(x-\mu \right)\right)}^{\beta }}$$

where Γ(·) denotes the Gamma function; $x,\mu \in R^{d}$ and μ are the mean vectors; Σ is a d×d real symmetrical positive definite matrix called the scatter matrix; and *m* and β>0 are the scale and shape parameters of the MGGD. The shape parameter controls the peak’s sharpness and tail extension in the probability density function. It is worth noting that when β=1, the MGGD becomes the multivariate Gaussian distribution. If β<1, the distribution will have a sharper peak and a larger tail. However, when β tends to infinity, MGGD is close to a multivariate uniform distribution [31].

### 2.1. Data Weighting

Recent research has shown that data weighting could improve modeling capabilities (see, for example, [44] and references therein). Here, we follow these works via an extension to GGMM-based modeling. As proposed in [44], for example, each sample obtains a corresponding weight *w* greater than 0. If we consider the likelihood for a specific sample *x*, we can write it as $p{\left(x;\mu ,\Sigma ,\beta ,m\right)}^{w}$. This is not a probability distribution because the integral is not equal to one. However, we notice that $p{\left(x;\mu ,\Sigma ,\beta ,m\right)}^{w}\propto p\left(x;\mu ,\Sigma ,\beta ,mw^{-1/\beta }\right)$, and thus we can then obtain the PDF with weight as a parameter:

(2) $$\hat{p}\left(x;\theta ,w\right)=p\left(x;\mu ,\Sigma ,\beta ,mw^{-1/\beta }\right)$$

where θ={μ,Σ,β,m}. It can be seen that individual weight directly influences *m* in the PDF here. Still, in parameter estimation, the weights will simultaneously affect the scale and shape in the final mixture. Having the new distribution in Equation (2) in hand, we can obtain the *K*-component mixture:

(3) $$\tilde{p}\left(x;\Theta ,w\right)=\sum_{k=1}^{K}\pi_{k}p\left(x;\mu_{k},\Sigma_{k},\beta_{k},m_{k}w^{-1/\beta_{k}}\right)$$

where $\Theta =\{\pi_{1},...,\pi_{K},\theta_{1},...,\theta_{K}\}$ denotes the parameter set of the model, and $\left(\pi_{1},...,\pi_{K}\right)$ are the mixing coefficients which satisfy $\pi_{k}>0$ and $\sum_{k=1}^{K}\pi_{k}=1$. $\theta_{k}=\left(\mu_{k},\Sigma_{k},\beta_{k},m_{k}\right)$ are the parameters of the $k^{th}$ component. Let $X=\{x_{1},...,x_{N}\}$ represent the whole data set and $W=\{w_{1},...,w_{N}\}$ is the weight set, then the log-likelihood function is given by:

(4) $$L\left(X|\Theta ,W\right)=\sum_{i=1}^{N}\ln\left[\sum_{k=1}^{K}\pi_{k}p\left(x_{i};\mu_{k},\Sigma_{k},\beta_{k},m_{k}w_{i}^{-1/\beta_{k}}\right)\right]$$

### 2.2. MGGD with Fixed Weights

For maximum likelihood estimation, the missing variables $Z=\{z_{1},...,z_{N}\}$ are introduced. If $x_{i}$ is generated by the *k*th component, then $z_{i}=k$. We assume that the weights are already known by prior knowledge, so the expected complete data log-likelihood (*Q* function) can be written as:

(5) $$\begin{array}{c} Q_{c}\left(\Theta ,\Theta^{\left(r\right)}\right)=E_{p\left(Z|X;W,Q^{\left(r\right)}\right)}\left[\ln P\left(X,Z;W,\Theta \right)\right]\\ \overset{\Theta }{=}\sum_{i=1}^{N}\sum_{k=1}^{K}\eta_{ik}[\ln\pi_{k}+\ln\beta_{k}+\frac{d}{2\beta_{k}}\ln w_{i}-\ln\Gamma \left(\frac{d}{2\beta_{k}}\right)-\frac{d}{2\beta_{k}}\ln2\\ -\frac{d}{2}\ln m_{k}-\frac{1}{2}\ln\left|\Sigma_{k}\right|-\frac{w_{i}{\left[{\left(x_{i}-\mu_{k}\right)}^{T}\Sigma_{k}^{-1}\left(x_{i}-\mu_{k}\right)\right]}^{\beta_{k}}}{2m_{k}^{\beta_{k}}}] \end{array}$$

where $E_{P}\left[\cdot \right]$ is the expectation with respect to the distribution *P* and $\overset{\Theta }{=}$ represents items only related to Θ. Subsequently, the optimal parameters $\Theta^{*}$ can be obtained by the EM algorithm. In the expectation step, the posteriors are updated with:

(6) $$\eta_{ik}=p\left(z_{i}=k|x_{i};\Theta ,w_{i}\right)=\frac{\hat{p}\left(x_{i};\theta_{k},w_{i}\right)}{\sum_{k=1}^{K}\pi_{k}\hat{p}\left(x_{i};\theta_{k},w_{i}\right)}$$

By taking the derivatives of the complete data log-likelihood with respect to the parameters and making the results equal to zero, we can obtain the parameter update formulas:

(7) $$\pi_{k}=\frac{1}{N}\sum_{i=1}^{N}\eta_{ik}$$

(8) $$\begin{array}{c} m_{k}={\left[\frac{\beta_{k}}{d\sum_{i=1}^{N}\eta_{ik}}\sum_{i=1}^{N}\eta_{ik}w_{i}{\left[{\left(x_{i}-\mu_{k}\right)}^{T}\Sigma_{k}^{-1}\left(x_{i}-\mu_{k}\right)\right]}^{\beta_{k}}\right]}^{\frac{1}{\beta_{k}}} \end{array}$$

(9) $$\mu_{k}=\frac{\sum_{i=1}^{N}\eta_{ik}w_{i}{\left[{\left(x_{i}-\mu_{k}\right)}^{T}\Sigma_{k}^{-1}\left(x_{i}-\mu_{k}\right)\right]}^{\beta_{k}-1}x_{i}}{\sum_{i=1}^{N}\eta_{ik}w_{i}{\left[{\left(x_{i}-\mu_{k}\right)}^{T}\Sigma_{k}^{-1}\left(x_{i}-\mu_{k}\right)\right]}^{\beta_{k}-1}}$$

(10) $$\Sigma_{k}=\frac{\beta_{k}}{m_{k}^{\beta_{k}}\sum_{i=1}^{N}\eta_{ik}}\sum_{i=1}^{N}\left[\frac{\eta_{ik}w_{i}\left(x_{i}-\mu_{k}\right){\left(x_{i}-\mu_{k}\right)}^{T}}{{\left[{\left(x_{i}-\mu_{k}\right)}^{T}\Sigma_{k}^{-1}\left(x_{i}-\mu_{k}\right)\right]}^{1-\beta_{k}}}\right]$$

After replacing $m_{k}$ in Equation (10) with the previous result in Equation (8), we can find that $\Sigma_{k}$ is the independent form $m_{k}$. If we let $y_{i}={\left(x_{i}-\mu_{k}\right)}^{T}\Sigma_{k}^{-1}\left(x_{i}-\mu_{k}\right)$, we have:

(11) $$\Sigma_{k}=\frac{d}{\sum_{i=1}^{N}\left(\eta_{ik}w_{i}y_{i}^{\beta_{k}}\right)}\sum_{i=1}^{N}\left[\frac{\eta_{ik}w_{i}\left(x_{i}-\mu_{k}\right){\left(x_{i}-\mu_{k}\right)}^{T}}{y_{i}^{1-\beta_{k}}}\right]$$

Furthermore, it is worth noting that $\mu_{k}$ and $\Sigma_{k}$ do not have closed solutions; they are both solved through the fixed point (FP) method. According to Banach’s fixed point theorem [45], if (S,d) is a non-empty complete metric space with a contraction mapping T:S→S, then there exists a unique fixed point $S^{*}$ in *S*. The authors in [31] proved the convergence of $\Sigma_{k}$ and explained the existence and uniqueness of the fixed point, based on the fact that β∈(0,1] and Σ is a positive definite real symmetric matrix. This is also consistent with our assumptions about β. We introduce the Frobenius norm defined in Equation (12) to measure the difference between $S_{n}$ and $S_{n-1}$ in the fixed point iteration. The process stops when the approximate solution satisfies the preset precision. Furthermore, to ensure the convergence of the FP equation, the value range of weights should also be between zero and one.

(12) $${∥S_{n}-S_{n-1}∥}_{F}=\sqrt{\sum_{i=1}^{m}\sum_{j=1}^{n}{\left|s_{n}^{\mathrm{ij}}-s_{n-1}^{ij}\right|}^{2}}$$

Then parameter β can be estimated using Newton–Raphson iterations [42,43,44]:

(13) $$\beta_{k}^{\left(t+1\right)}=\beta_{k}^{\left(t\right)}-\xi \frac{f\left(\beta_{k}^{\left(t\right)}\right)}{f^{\prime }\left(\beta_{k}^{\left(t\right)}\right)}$$

where ξ is the learning rate used to prevent the oscillation and overflow in the iterative process, and it is usually around 0.1. If necessary, the method of exponential decay can be applied to make the convergence more stable. $f(\beta_{k}^{\left(t\right)})$ and $f^{\prime }\left(\beta_{k}^{\left(t\right)}\right)$ are given as follows:

(14) $$\begin{array}{c} f\left(\beta_{k}\right)=\frac{d\sum_{i=1}^{N}\eta_{ik}}{2\sum_{i=1}^{N}\left(\eta_{ik}w_{i}y_{i}^{\beta_{k}}\right)}\sum_{i=1}^{N}\left[\eta_{ik}w_{i}y_{i}^{\beta_{k}}\ln\left(y_{i}\right)\right]\\ +\frac{d\sum_{i=1}^{N}\left(\eta_{ik}\ln w_{i}\right)}{2\beta_{k}}-\frac{d\sum_{i=1}^{N}\eta_{ik}[}{2\beta_{k}}\left[\Psi \left(\frac{d}{2\beta_{k}}\right)+\ln2\right]\\ -\sum_{i=1}^{N}\eta_{ik}-\frac{d\sum_{i=1}^{N}\eta_{ik}}{2\beta_{k}}\ln\left[\frac{\beta_{k}}{d\sum_{i=1}^{N}\eta_{ik}}\sum_{i=1}^{N}\left(\eta_{ik}w_{i}y_{i}^{\beta_{k}}\right)\right] \end{array}$$

(15) $$\begin{array}{c} f^{\prime }\left(\beta_{k}\right)=\frac{d\sum_{i=1}^{N}\eta_{ik}}{2{\left(\sum_{i=1}^{N}\eta_{ik}w_{i}y_{i}^{\beta_{k}}\right)}^{2}}[\left(\sum_{i=1}^{N}\eta_{ik}w_{i}y_{i}^{\beta_{k}}\right)\\ \left(\sum_{i=1}^{N}\eta_{ik}w_{i}y_{i}^{\beta_{k}}{\left(\ln y_{i}\right)}^{2}\right)-{\left(\sum_{i=1}^{N}\eta_{ik}w_{i}y_{i}^{\beta_{k}}\ln y_{i}\right)}^{2}]\\ -\frac{d\sum_{i=1}^{N}\left(\eta_{ik}\ln w_{i}\right)}{2\beta_{k}^{2}}+\frac{d\sum_{i=1}^{N}\eta_{ik}}{2\beta_{k}^{2}}\left[\Psi \left(\frac{d}{2\beta_{k}}\right)+\ln2\right]\\ +\frac{d^{2}\sum_{i=1}^{N}\eta_{ik}}{4\beta_{k}^{3}}\Psi^{\prime }\left(\frac{d}{2\beta_{k}}\right)+\frac{d\sum_{i=1}^{N}\eta_{ik}}{2\beta_{k}^{2}}[\ln\left(\frac{\beta_{k}}{dN}\right)\\ +\ln\left(\sum_{i=1}^{N}\eta_{ik}w_{i}y_{i}^{\beta_{k}}\right)-1-\beta_{k}\frac{\sum_{i=1}^{N}\eta_{ik}w_{i}y_{i}^{\beta_{k}}\ln y_{i}}{\sum_{i=1}^{N}\eta_{ik}w_{i}y_{i}^{\beta_{k}}}] \end{array}$$

where Ψ(·) is the digamma function.

### 2.3. Weights Considered as Random Variables

Above, we have derived the WMGGMM parameter updating formulas with fixed weights. However, it is pointed out in [44] that the Bayesian formalism is more inclined to treat parameters as random variables and update the posterior of parameters by combining the parameters prior with the observed sample. Under this framework, the limitation of insufficient prior knowledge on the accuracy of weights is reduced, and the inference process of weights will also be more explanatory. As mentioned before, the generalized Gaussian distribution belongs to the family of elliptic distributions, and thus we select the same prior distribution, i.e., Gamma distribution, as in [44]. At this point, the prior and posterior of the parameters have the same form. The advantage is that when we make a new observation, we do not have to re-calculate the whole process but only directly obtain the posterior distribution through the parameters, which undoubtedly simplifies the updating process of weight parameters. Then, the posterior will become the prior in the next calculation, Therefore, we can obtain:

(16) $$p\left(w;\phi \right)=G\left(w;a,b\right)=\Gamma {\left(a\right)}^{-1}b^{a}w^{a-1}e^{-bw}$$

where G(w;a,b) is the Gamma distribution, and ϕ={a,b} are the parameters of the prior distribution of *w*. The mean and variance of random variable *w* are given by:

(17) E[w]=a/b

(18) $$Var\left[w\right]=a/b^{2}$$

Due to the addition of prior parameters, the log-likelihood of the complete data becomes the following form:

(19) $$Q_{c}\left(\Theta ,\Theta^{\left(r\right)}\right)=E_{p\left(Z,W|X;\Theta^{\left(r\right)},\Phi \right)}\left[\ln P\left(X,Z,W;\Theta ,\Phi \right)\right]$$

where $\Phi =\{\phi_{1},...,\phi_{N}\}$ and $\phi_{i}=\left\{a_{i},b_{i}\right\}$. The posterior distribution factorizes on *i* as follows:

(20) $$P\left(Z,W|X;\Theta^{\left(r\right)},\Phi \right)=\prod_{i=1}^{N}p\left(z_{i},w_{i}|x_{i};\Theta^{\left(r\right)},\phi_{i}\right)$$

Each of these product terms can be expressed as two-factor expressions:

(21) $$\begin{array}{c} p\left(z_{i},w_{i}|x_{i};\Theta^{\left(r\right)},\phi_{i}\right)=\\ p\left(w_{i}|z_{i},x_{i};\Theta^{\left(r\right)},\phi_{i}\right)p\left(z_{i}|x_{i};\Theta^{\left(r\right)},\phi_{i}\right) \end{array}$$

According to the above formula, the expectation step in the EM algorithm is divided into two parts (E-Z step and E-W step). In the E-Z step, the marginal posterior distribution of $z_{i}$ is obtained by integrating over $w_{i}$:

(22) $$\begin{array}{cc} \eta_{ik} & =\int p\left(z_{i}=k,w_{i}|x_{i};\Theta^{\left(r\right)},\phi_{i}\right)dw_{i}\\ & \overset{k}{\propto }\int \pi_{k}p\left(x_{i}|z_{i}=k,w_{i};\Theta^{\left(r\right)}\right)p\left(w_{i};\phi_{i}\right)dw_{i}\\ & =\int \pi_{k}\hat{p}\left(x_{i};\theta_{k},w_{i}\right)G\left(w_{i};a_{i},b_{i}\right)dw_{i}\\ & \propto \pi_{k}\bar{p}\left(x_{i};\mu_{k},\Sigma_{k},\beta_{k},m_{k},a_{i},b_{i}\right) \end{array}$$

where $\bar{p}\left(x;\mu ,\Sigma ,\beta ,m,a,b\right)$ is given as:

(23) $$\begin{array}{c} \bar{p}\left(x;\mu ,\Sigma ,\beta ,m,a,b\right)=\\ \frac{\beta \Gamma \left(\frac{d}{2}\right)\Gamma \left(a+\frac{d}{2\beta }\right)}{{\left(m\pi \right)}^{\frac{d}{2}}{\left(2b\right)}^{\frac{d}{2\beta }}{\left|\Sigma \right|}^{\frac{1}{2}}\Gamma \left(a\right)\Gamma \left(\frac{d}{2\beta }\right)}{\left[\frac{{\left({\left(x-\mu \right)}^{T}\Sigma^{-1}\left(x-\mu \right)\right)}^{\beta }}{2bm^{\beta }}+1\right]}^{-\left(a+\frac{d}{2\beta }\right)} \end{array}$$

In step E-W, according to the property of conjugate distribution, we can obtain:

(24) $$\begin{array}{c} p\left(w_{i}|z_{i}=k,x_{i};\Theta ,\phi_{i}\right)\overset{w_{i}}{\propto }p\left(x_{i}|z_{i}=k,w_{i};\Theta \right)p\left(w_{i};\phi_{i}\right)\\ =\hat{p}\left(x_{i};\theta_{k},w_{i}\right)G\left(w_{i};a_{i},b_{i}\right)=G\left(w_{i};a_{i}^{\left(r+1\right)},b_{i}^{\left(r+1\right)}\right) \end{array}$$

Thus, the updating formulas of prior parameters can be obtained:

(25) $$a_{ik}^{\left(r+1\right)}=a_{i}^{\left(0\right)}+\frac{d}{2\beta_{k}}$$

(26) $$b_{ik}^{\left(r+1\right)}=b_{i}^{\left(0\right)}+\frac{{\left[{\left(x_{i}-\mu_{k}\right)}^{T}\Sigma_{k}^{-1}\left(x_{i}-\mu_{k}\right)\right]}^{\beta_{k}}}{2m_{k}^{\beta_{k}}}$$

(27) $${\bar{w}}_{ik}=E_{P(w_{i}|z_{i}=k,x_{i},\Theta^{\left(r\right)},\phi_{i})}\left[w_{i}\right]=\frac{a_{ik}^{\left(r+1\right)}}{b_{ik}^{\left(r+1\right)}}$$

This explains the outliers shielding feature of the weighted algorithm. As an outlier is by definition far from the center of all components, it has a low posterior weight ${\bar{w}}_{ik}$ for each component and then a low mean posterior probability ${\bar{w}}_{i}$. By expanding Equation (19), we can obtain a result similar to the *Q* function with fixed weights:

(28) $$\begin{array}{c} Q_{c}\left(\Theta ,\Theta^{\left(r\right)}\right)=\\ \sum_{i=1}^{N}\sum_{k=1}^{K}\int_{w_{i}}\eta_{ik}\ln\pi_{k}\hat{p}\left(x_{i};\theta_{k},w_{i}\right)\times p\left(w_{i}|x_{i},z_{i}=k,\Theta^{\left(r\right)},\phi_{i}\right)dw_{i}\\ \overset{\Theta }{=}\sum_{i=1}^{N}\sum_{k=1}^{K}\eta_{ik}[\ln\pi_{k}+\ln\beta_{k}+\frac{d}{2\beta_{k}}\ln{\bar{w}}_{ik}^{\left(r+1\right)}-\ln\Gamma \left(\frac{d}{2\beta_{k}}\right)-\frac{d}{2\beta_{k}}\ln2\\ -\frac{d}{2}\ln m_{k}-\frac{1}{2}\ln\left|\Sigma_{k}\right|-\frac{{\bar{w}}_{ik}^{\left(r+1\right)}{\left[{\left(x_{i}-\mu_{k}\right)}^{T}\Sigma_{k}^{-1}\left(x_{i}-\mu_{k}\right)\right]}^{\beta_{k}}}{2m_{k}^{\beta_{k}}}] \end{array}$$

Therefore, $w_{i}$ will be replaced with ${\bar{w}}_{ik}$ in all the parameter updates formulas of the mixture model. Since the equations are very similar, they are not repeated here.

### 2.4. Automatic Determination of the Number of Components

The model selection problem is tackled using the minimum message length (MML) criterion as proposed in [46]:

(29) $$\begin{array}{c} \Theta_{MML}=\underset{\Theta }{\arg\min}\{-\log P\left(\Theta \right)-Q_{R}\left(\Theta ,\Theta^{\left(r+1\right)}\right)\\ +\frac{1}{2}\log\left|I_{c}\left(\Theta \right)\right|+\frac{D(\Theta )}{2}\left(1+\log\frac{1}{12}\right)\} \end{array}$$

where $I_{C}\left(\Theta \right)$ denotes the expected complete Fisher information matrix (FIM) and D(Θ) is the dimensionality of the model. Using a similar process as in [46], we can show that

(30) $$\begin{array}{c} \Theta_{MML}=\underset{\Theta }{\arg\min}\{\frac{M}{2}\sum_{k\in K^{+}}\log\pi_{k}-Q_{R}\left(\Theta ,\Theta^{\left(r+1\right)}\right)\\ +\frac{K^{+}\left(M+1\right)}{2}\left(1+\log\frac{n}{12}\right)\} \end{array}$$

where $K^{+}$ is the set of non-empty components and $K^{+}$ is the number of elements in it. Moreover, we can rewrite the formula for calculating $\pi_{k}$ in the maximization step of the EM algorithm:

(31) $$\pi_{k}=\frac{\max\left(0,\sum_{i=1}^{N}\eta_{ik}-MK^{+}/2\right)}{\sum_{l=1}^{K}\max\left(0,\sum_{i=1}^{N}\eta_{il}-MK^{+}/2\right)}$$

The threshold for minimum support is high when the number of non-empty components is large at the beginning. Some components can be removed quickly. In the process of updating components one by one, the threshold gradually approaches the situation in [46] as the number of non-empty components decreases.

### 2.5. Complete Algorithm

The complete steps are outlined in Algorithm 1. We use k-means to initialize $\pi_{k},\mu_{k},\Sigma_{k}$. The initial parameter $\beta_{k}$ is specified as 0.5, and the parameter $m_{k}$ is calculated according to the pre-clustering results and the formula in [31]:

(32) $$m_{k}={\left\{\frac{\beta_{k}}{dN_{k}}\sum_{i=1}^{N_{k}}{\left[{\left(x_{i}-\mu_{k}\right)}^{T}\Sigma_{k}^{-1}\left(x_{i}-\mu_{k}\right)\right]}^{\beta_{k}}\right\}}^{\frac{1}{\beta_{k}}}$$

where $x_{i}\in X_{k}$ is the *i*th sample for the *k*th cluster, and $N_{k}$ is the number of samples.

We adopt the same data similarity measurement method based on the Gaussian kernel as in [44] for the initialization of weights. However, due to the constraint of the weight range, we modify it as follows:

(33) $$w_{i}=\frac{1}{q}\sum_{j\in S_{i}^{q}}exp\left[-\frac{d^{2}\left(x_{i},x_{j}\right)}{\sigma }\right]$$

where $d^{2}\left(x_{i},x_{j}\right)$ denotes the Euclidean distance, $S_{i}^{q}$ is the set containing q nearest neighbors of $x_{i}$, and σ is a positive scale. The default setting for q is 20. We can then calculate the initialization of the prior parameters of the weight through Equations (17) and (18), i.e., $a_{i}=w_{i}^{2}$ and $b_{i}=w_{i}$.

**Figure 1:**
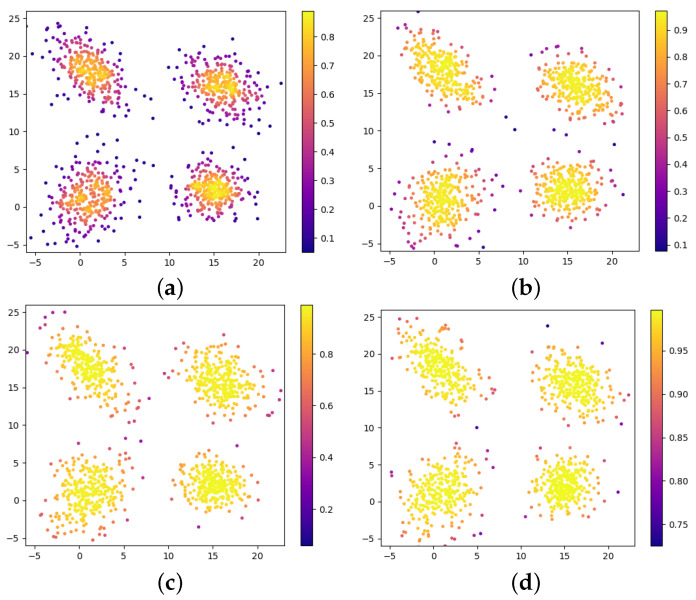
The influence of different Gaussian kernel parameter σ on the weights. (**a**) σ=1; (**b**) σ=5; (**c**) σ=10; (**d**) σ=50.

Figure 1 shows the impact of different σ on the weights; its significance is to change the degree of differentiation of the weights. When there is a small σ, the difference in the weights between dense and sparse areas becomes more pronounced. Conversely, the distribution of weights will be relatively close. In other words, a smaller σ is more beneficial at removing outliers and noise. However, it is important to note that when the σ is too small, the weight operation is equivalent to removing most of the points that are away from the clustering center in the data distribution. The actual points involved in parameter identification are reduced, leading to inadequate support of the components. Furthermore, the resulting mixed model parameters will have a large deviation from the original data distribution. Therefore, the selection of σ is a balance between model robustness and model accuracy. We set it to 25 in our case.
**Algorithm 1** Proposed WMGGMM algorithm with component-wise EM procedure.**Input:** $X={\left\{x_{i}\right\}}_{i=1}^{N}$; $\Phi^{\left(0\right)}={\left\{a_{i}^{\left(0\right)},b_{i}^{\left(0\right)}\right\}}_{i=1}^{N}$; $K_{max}$ $\Theta^{\left(0\right)}={\left\{\pi_{k}^{\left(0\right)},\mu_{k}^{\left(0\right)},\Sigma_{k}^{\left(0\right)},\beta_{k}^{\left(0\right)},m_{k}^{\left(0\right)}\right\}}_{k=1}^{K_{max}}$;**Output:** Optimal mixture model parameters: $\Theta^{*}$ Set: r=0, MML=+∞ **repeat**      **for** k=1 To $K_{max}$ **do**            E-Z step using (22):            $\eta_{ik}^{\left(r+1\right)}=\frac{\pi_{k}^{\left(r\right)}\bar{p}\left(x_{i};\mu_{k}^{\left(r\right)},\Sigma_{k}^{\left(r\right)},\beta_{k}^{\left(r\right)},m_{k}^{\left(r\right)},a_{ik}^{\left(r\right)},b_{ik}^{\left(r\right)}\right)}{\sum_{l=1}^{k_{max}}\pi_{l}^{\left(r\right)}\bar{p}\left(x_{i};\mu_{l}^{\left(r\right)},\Sigma_{l}^{\left(r\right)},\beta_{l}^{\left(r\right)},m_{l}^{\left(r\right)},a_{il}^{\left(r\right)},b_{il}^{\left(r\right)}\right)}$            Compute the # of non-empty components: $K^{+}$            E-W step using (25)–(27):                 $a_{ik}^{\left(r+1\right)}=a_{i}^{\left(0\right)}+\frac{d}{2\beta_{k}}$                 $b_{ik}^{\left(r+1\right)}=b_{i}^{\left(0\right)}+\frac{{\left[{\left(x_{i}-\mu_{k}\right)}^{T}\Sigma_{k}^{-1}\left(x_{i}-\mu_{k}\right)\right]}^{\beta_{k}}}{2m_{k}^{\beta_{k}}}$                 ${\bar{w}}_{ik}=a_{ik}^{\left(r+1\right)}/b_{ik}^{\left(r+1\right)}$             M step:                 $\pi_{k}^{\left(r+1\right)}=\frac{max(0,\eta_{ik}^{\left(r+1\right)}-K^{+}M/2)}{\sum_{l=1}^{K_{max}}max\left(0,\eta_{il}^{\left(r+1\right)}-K^{+}M/2\right)}$             **if** $\pi_{k}^{\left(r+1\right)}>0$ **then**                 Update $\mu_{k}$ using (9):                  **repeat**                      $\mu_{k}^{new}=T\left(\mu_{k}^{old}\right)$                  **until** $∥\mu_{k}^{new}-\mu_{k}^{old}{∥}_{F}<\epsilon$                  $\mu_{k}^{\left(r+1\right)}=\mu_{k}^{new}$                  Update $\Sigma_{k}$ using (11):                  **repeat**                      $\Sigma_{k}^{new}=T\left(\Sigma_{k}^{old}\right)$                  **until** $∥\Sigma_{k}^{new}-\Sigma_{k}^{old}{∥}_{F}<\epsilon$                  $\Sigma_{k}^{\left(r+1\right)}=\Sigma_{k}^{new}$                  Update $\beta_{k}$ using (13)–(15):                  **repeat**                      $\beta_{k}^{new}=\beta_{k}^{old}-\zeta \frac{f(\beta_{k}^{old})}{f^{\prime }\left(\beta_{k}^{old}\right)}$                  **until** $|\mu_{k}^{new}-\mu_{k}^{old}|<\epsilon$                   $\beta_{k}^{\left(r+1\right)}=\beta_{k}^{new}$                  Update $m_{k}^{\left(r+1\right)}$ using (8)            **end if**        **end for**        Compute $MML^{\left(r+1\right)}$ using (30)        r=r+1    **until** $|\Delta MML^{\left(r\right)}|<\epsilon$    Return the parameters Θ of non-empty components as optimal mixture model parameters $\Theta^{*}$

## 3. Experimental Results

### 3.1. Synthetic Data

First, we demonstrate the performance of the WMGGMM model through synthetic data. The method for generating data points that follow an MGGD comes from [31]:

(34) $$x\in R^{d}\tilde{=}\tau C^{1/2}u$$

where x is a random vector that follows an MGGD with scatter matrix C=mΣ and shape parameter β, and $\tilde{=}$ denotes equivalence on the distribution; x is a random vector uniformly distributed on a unit sphere and τ is a positive scalar random variable that satisfies $\tau^{2\beta }∼\Gamma \left(d/(2\beta ),2\right)$.

Table 1 and Table 2 show the parameter estimation results for two generated two-dimensional data sets from the 4- and 3-component mixture models, respectively, where $\rho_{k}=cov\left(X,Y\right)/$$\sqrt{var(X)var(Y)}$ represents the correlation coefficient used to measure the slope of the scatter matrix.

We can see that the estimated parameters are close to the real ones. According to the correlation coefficient comparisons, the slopes of the real and estimated scatter matrices are also close. Due to the role of weight, the points essentially involved in parameter estimation are concentrated in data-intensive areas, leading to a reduced scale parameter *m*. However, the change in parameter β is not necessarily a decrease. Suppose that β is small in the default setting. In the big trailing part, the data points are distributed sparsely. The weight operation will remove most of these points, so the shape of data is changed, and the final estimated β will become larger instead.

**Figure 2:**
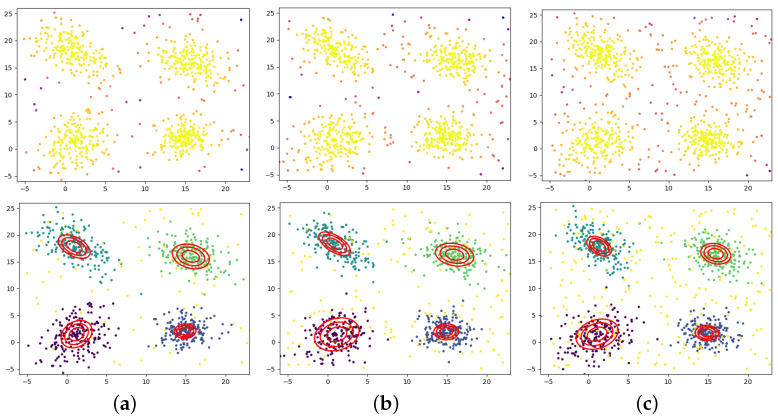
Estimation results of the mixture model parameters with different noise levels: (**a**) 10% noise, (**b**) 20% noise, (**c**) 30% noise.

Figure 2 presents the parameter estimation results at different noise levels. Parameter estimation is carried out after the proportional addition of random uniform noise in the primary distribution area ([−5.25, 5.25]) of the original data. When σ is appropriately selected, the weights remove most of the noise and outliers, and the actual points involved in parameter estimation are mostly in the desired original data distribution. The final results show that the mixture models of the three different cases are very similar, which effectively proves the WMGGMM algorithm’s robustness.

**Figure 3:**
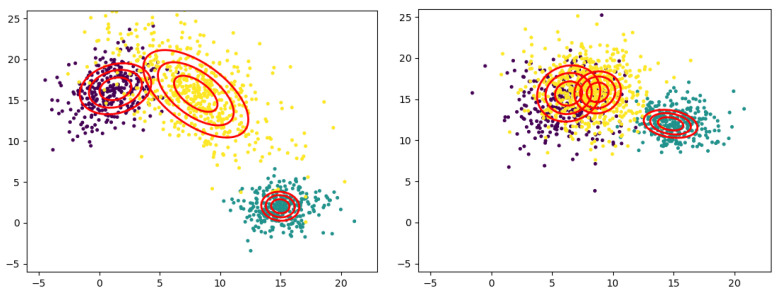
The results of parameter estimation in the case of component overlap.

The results of the mixture model for overlapping data are shown in Figure 3. The introduction of shape parameter β enables MGGD to improve the identification ability of data distribution peaks. Although the overall distribution of two components overlap, the WMGGMM algorithm can still accurately identify the parameters of each component when their centers do not coincide.

**Figure 4:**
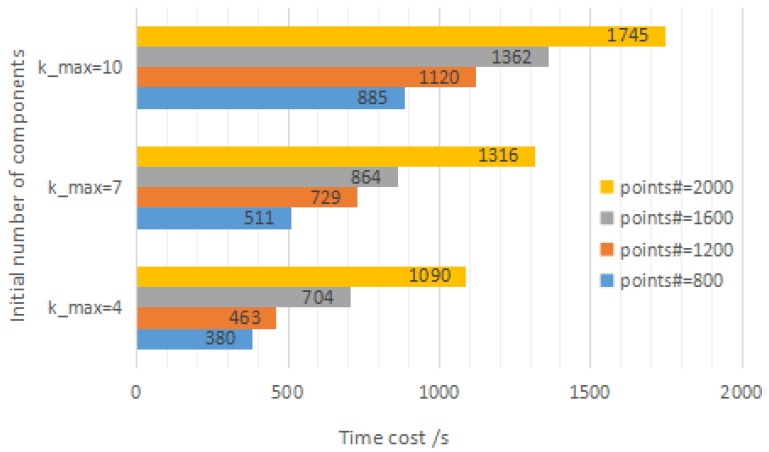
Performance analysis of WMGGMM for 2-D data.

Figure 4 displays the execution time of the WMGGMM algorithm under different conditions (the default number of components is four). The average value of five independent running times is taken as the final result, and the unit is seconds. Because most of the algorithm’s parameters need to be estimated iteratively (fixed-point equations and Newton–Raphson method), the algorithm’s running time is much higher than that of the standard EM algorithm. The anti-interference performance of the model is improved by sacrificing some efficiency. The time here can only be used as a relative reference since the algorithm’s run time is influenced by computer performance, hardware and software acceleration, and randomness of parameter initialization.

### 3.2. Point Cloud Registration Using WMGGMM

We consider the approach proposed in [23], in the case of GMM, to perform WMGGMM-based registration. Two mixture models are adopted to describe the target scene and the scene to be registered. Then, the difference between these two models is applied to update the registration parameters. Nevertheless, the algorithm proposed in [23] is still a method based on point to point in essence. The data pre-clustering is not considered, but each point in the data set is used as a mean to initialize a mixture component. This approach is equivalent to converting the entire data set into a mixture model with multiple simple components. However, this method of initializing the mixture model is not suitable in the point cloud with a large data volume. The subsequent L2 divergence simplifies the derivation based on the transformation model. However, the derivative optimization process is closely related to the expression of the specific model. If the transformation model is changed, all optimization processes need to be re-derived. Therefore, we propose a method based on WMGGMM to pre-cluster the target scene and the scene to be registered and extract the key features to form the mixture models. Then, the optimal registration parameters are found by using stochastic optimization through the KLD between the mixture models. The KLD of the two statistical models is defined as follows:

(35) $$KLD\left(f||g\right)=\int_{x}f\left(x\right)\log\frac{f(x)}{g(x)}dx$$

Unfortunately, the KLD between the mixture models has no analytical expression, so several approximations have been proposed [47,48,49]. Compared with GMM, the KLD of the generalized Gaussian mixture model using the inner product of the components is too complicated due to the introduction of shape parameters. Even though a method for calculating the KLD of two MGGDs is proposed in [38], the matched bound and variational approximations are also unfeasible because the premise of this approach is that all MGGDs have a zero mean. However, the mean value of components is significant in the point cloud registration scenario. Therefore, we finally use Monte Carlo sampling to calculate the KLD between two GGMMs:

(36) $$KLD_{MC}(P_{S}\left|\right|P_{M})=\frac{1}{n}\sum_{i=1}^{n}\left[\log P_{S}\left(x_{i}\right)-\log P_{M}\left(x_{i}\right)\right]$$

where $P_{S}\left(x\right)$ and $P_{M}\left(x\right)$ are the probability density functions of the GGMM obtained from the target scene and the scene to be registered, respectively, and ${\left\{x_{i}\right\}}_{i=1}^{n}$ denotes *n* samples taken from $P_{S}\left(x\right)$. In the above formula, the sum of the probability of samples replaces the integral, and the Monte Carlo method is the only method that can approximate the actual value of KLD when there are enough sample points. The Gibbs sampling, one of the Markov Chain Monte Carlo (MCMC) sampling techniques, is applied in this process using n=1000.

We assume that the point cloud transformation model is:

(37) $$X=f_{T}\left(X_{0},\Omega \right)$$

where $X_{0}$ and *X* are the point sets before and after the transformation, and $\Omega =\{\omega_{1},...,\omega_{m}\}$ is the parameter set of the transformation model. Then, we can express the point cloud registration problem in the following form:

(38) $$\Omega^{*}=\underset{\Omega }{\arg\min}\left\{KLD_{MC}\left[\mathrm{ggmm}\left(S\right)∥\mathrm{ggmm}\left(f_{T}\left(M,\Omega \right)\right)\right]\right\}$$

where S is the target scene point set, M is the point set to be registered, and ggmm(·) denotes the obtained PDF of the GGMM from a data set using the WMGGMM algorithm. The complete point cloud registration algorithm using WMGGMM is shown in Algorithm 2. However, it is only a general framework and does not specify a concrete random optimization method. The stochastic optimization technique can be selected depending on needs, as long as the definition of the loss function in (38) is satisfied, and the optimization domain is Ω. The simulated annealing (SA) algorithm is used in this paper.
**Algorithm 2** Point cloud registration algorithm based on WMGGMM and stochastic optimization.**Input:** Scene set: *S*, Model set: *M*, Initial transformation parameter set: $\Omega^{\left(0\right)}$**Output:** Optimal transformation parameter set: $\Omega^{*}$    Set: $KLD^{old}=0$, $KLD^{new}=0$, $\Omega^{old}=\Omega^{\left(0\right)}$    $ggmm_{s}=GGMM\left(S\right)$    Gibbs sampling on $ggmm_{s}$ to get: $X_{sample}={\left\{x_{i}\right\}}_{i=1}^{1000}$    **repeat**        $ggmm_{m}^{old}=ggmm\left(f_{T}\left(M,\Omega^{old}\right)\right)$        $KLD^{old}=KLD_{MC}(ggmm_{s}||ggmm_{m}^{old})$        $\Omega^{new}=RandomGeneration\left(\Omega^{old}\right)$        $ggmm_{m}=ggmm\left(f_{T}\left(M,\Omega^{new}\right)\right)$        $KLD^{new}=KLD_{MC}(ggmm_{s}||ggmm_{m}^{new})$        **if** $KLD^{new}<KLD^{old}$ **then**           $\Omega^{old}=\Omega^{new}$        **end if**     **until** some stopping criterion is satisfied     Return $\Omega^{*}=\Omega^{old}$

When the transformation model is rigid, i.e., $X=R_{\alpha }X_{0}+t$, the optimization process can be further simplified, where $R_{\alpha }$ is the rotation matrix with an angle of α and *t* is a translation vector. The transformation of the data set is equivalent to the rigid change of the corresponding mixture model. We can obtain $\mu_{k}^{\prime }=R_{\alpha }\mu_{k}+t$ and $\Sigma_{k}^{\prime }=R_{\alpha }^{T}\Sigma_{k}R_{\alpha }$; the shape parameter β and scale parameter *m* are not affected during the transformation. In other words, we only calculate the mixture models of the target scene and the scene to be registered through WMGGMM at the beginning, and the parameter estimation does not need to be repeated during optimization. The transformation models used in the experiments below are all rigid transformations and all test data are generated based on the method in the previous section.

**Figure 5:**
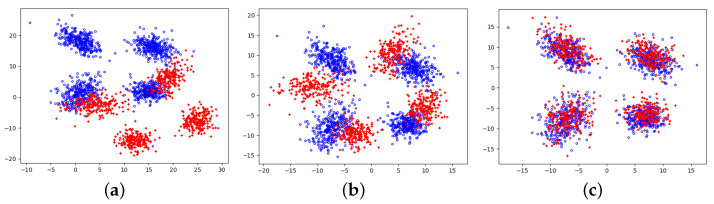
Registration results of point clouds with different sampling rates. (**a**) original; (**b**) centralized; (**c**) registration.

The unlikeness in sampling rate is a common challenge in point cloud registration. In general, the original scene will have a higher sampling rate to achieve more accurate modeling. In comparison, the scene to be registered may have a lower sampling rate due to the limitations of the sampling environment and tasks. Figure 5 shows the registration results of point sets with different sampling rates. The target scene (blue) has 1200 samples, while the scene to be registered (red) has 800. Although the sampling rate varies, the two scenes’ data meet similar statistical distributions, so the method based on GGMM can still effectively register the two sets with rotational and translational errors of 1.5% and 4.3%, respectively.

**Figure 6:**
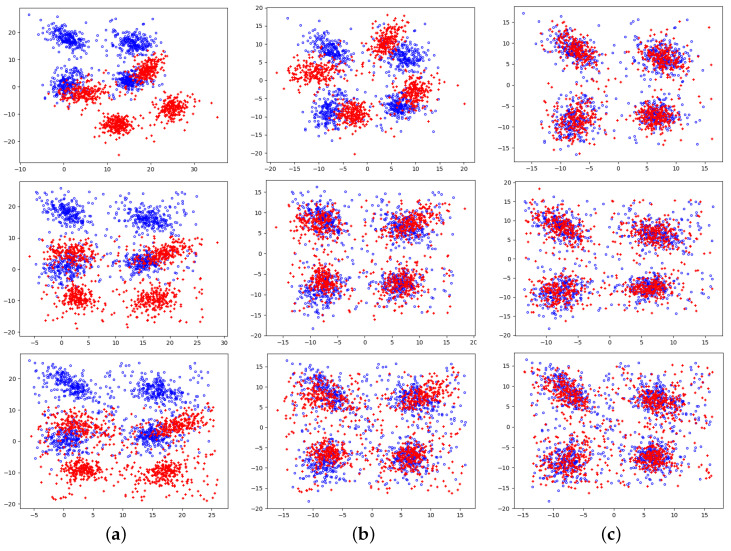
Registration results with 10% (top), 20% (middle), and 30% (bottom) noise levels. (**a**) original; (**b**) centralized; (**c**) registration.

The registration results at different noise levels are given in Figure 6. The outcomes show that our method can effectively remove the noise and outliers’ interference and extract the main features of the point cloud for matching, and the algorithm has good robustness with average rotational and translational errors of 3.5% and 6.3%, respectively.

**Figure 7:**
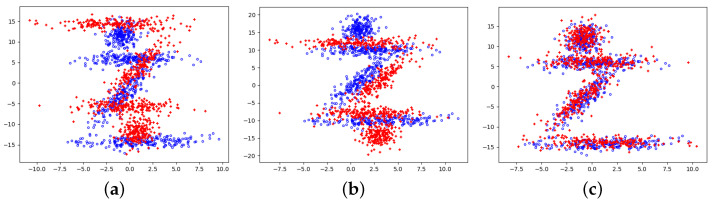
The registration results when locally optimal solution exists. (**a**) original; (**b**) centralized; (**c**) registration.

In Figure 7, we designed a point cloud shaped like the Chinese character “zhi” with a symmetrical approximate center (rotational and translational errors of 6.1% and 9.3%, respectively). The distinction between it and the original graph after 180 degrees of rotation is only at one “point”. Hence, it is straightforward to fall into the optimal local solution in registration optimization. However, in several experiments, only 6% fell into local optima. Compared with derivative optimization, stochastic optimization provides the ability to jump out of the local optimal solution.

## 4. Conclusions

This paper proposes a weighted multivariate generalized Gaussian mixture model and combines it with the stochastic optimization algorithm for point cloud rigid registration. This method requires enough samples in the registration scene to meet the minimum support of components. It extracts the data’s mass features rather than the edge features (contour and shell), so it is suitable for substantial point clouds with a large data volume. The introduction of weights and the ability of generalized Gaussian distributions to describe peak data can effectively reduce the influence of noise and outliers and obtain the critical features of data-intensive regions. Experimental results attest that the algorithm has sufficient robustness. The stochastic optimization algorithm reduces the coupling between algorithm modules, intensifies the algorithm’s expansibility, and provides a more potent global optimization capability. However, in the mixture model’s parameter estimation process, almost all parameters need to be learned iteratively; therefore, some performance is sacrificed to enhance the algorithm’s accuracy. In addition, this work only considered a 2D scenario. Future work will be committed to improve the parameter estimation approach to improve the algorithm’s performance and extend the approach to 3D scenarios.

## Figures and Tables

**Table 1:** Estimation of the mixture model parameters for the first synthetic data set.

Default Mixture Model Parameters				
$\pi_{k}$	$\mu_{k}$	$\beta_{k}$	$C_{k}$	$\rho_{k}$
0.25 (N = 300)	$\left[\begin{array}{cc} 1 & 1 \end{array}\right]$	0.85	$\left[\begin{array}{cc} 3 & 1\\ 1 & 5 \end{array}\right]$	0.35
0.25 (N = 300)	$\left[\begin{array}{cc} 15 & 2 \end{array}\right]$	0.85	$\left[\begin{array}{cc} 2 & 0\\ 0 & 2 \end{array}\right]$	0.00
0.25 (N = 300)	$\left[\begin{array}{cc} 1 & 18 \end{array}\right]$	0.85	$\left[\begin{array}{cc} 3 & -2\\ -2 & 4 \end{array}\right]$	−0.58
0.25 (N = 300)	$\left[\begin{array}{cc} 16 & 16 \end{array}\right]$	0.85	$\left[\begin{array}{cc} 3 & -1\\ -1 & 3 \end{array}\right]$	−0.33
Estimated mixture model parameters				
$\pi_{k}$	$\mu_{k}$	$\beta_{k}$	$C_{k}$	$\rho_{k}$
0.2444	$\left[\begin{array}{cc} 0.98 & 0.90 \end{array}\right]$	0.72	$\left[\begin{array}{cc} 1.43 & 0.57\\ 0.57 & 2.94 \end{array}\right]$	0.28
0.2586	$\left[\begin{array}{cc} 15.11 & 2.05 \end{array}\right]$	0.70	$\left[\begin{array}{cc} 0.60 & -0.04\\ -0.04 & 0.67 \end{array}\right]$	−0.06
0.2482	$\left[\begin{array}{cc} 1.15 & 17.99 \end{array}\right]$	0.75	$\left[\begin{array}{cc} 1.39 & -1.00\\ -1.00 & 2.15 \end{array}\right]$	−0.58
0.2486	$\left[\begin{array}{cc} 15.98 & 15.99 \end{array}\right]$	0.77	$\left[\begin{array}{cc} 1.12 & -0.3\\ -0.3 & 1.05 \end{array}\right]$	−0.27

**Table 2:** Estimation of the mixture model parameters for the second synthetic data set.

Default Mixture Model Parameters				
$\pi_{k}$	$\mu_{k}$	$\beta_{k}$	$C_{k}$	$\rho_{k}$
0.25 (N = 300)	$\left[\begin{array}{cc} 8 & 16 \end{array}\right]$	0.60	$\left[\begin{array}{cc} 3 & -2\\ -2 & 4 \end{array}\right]$	−0.58
0.25 (N = 300)	$\left[\begin{array}{cc} 15 & 2 \end{array}\right]$	0.85	$\left[\begin{array}{cc} 2 & 0\\ 0 & 2 \end{array}\right]$	0.00
0.50 (N = 600)	$\left[\begin{array}{cc} 1 & 3 \end{array}\right]$	0.85	$\left[\begin{array}{cc} 3 & 1\\ 1 & 5 \end{array}\right]$	0.35
Estimated mixture model parameters				
$\pi_{k}$	$\mu_{k}$	$\beta_{k}$	$C_{k}$	$\rho_{k}$
0.2596	$\left[\begin{array}{cc} 7.89 & 16.05 \end{array}\right]$	0.76	$\left[\begin{array}{cc} 2.27 & -1.34\\ -1.34 & 3.39 \end{array}\right]$	−0.48
0.2817	$\left[\begin{array}{cc} 14.90 & 2.12 \end{array}\right]$	0.67	$\left[\begin{array}{cc} 0.62 & -0.17\\ -0.17 & 0.78 \end{array}\right]$	−0.24
0.4587	$\left[\begin{array}{cc} 0.98 & 3.11 \end{array}\right]$	0.70	$\left[\begin{array}{cc} 0.91 & 0.61\\ 0.61 & 2.12 \end{array}\right]$	0.44

## Data Availability

Not applicable.

## References

[1] Zhu H., Guo B., Zou K., Li Y., Yuen K.V., Mihaylova L., Leung H. (2019). A review of point set registration: From pairwise registration to groupwise registration. Sensors.

[2] Hirose O. (2021). A Bayesian Formulation of Coherent Point Drift. IEEE Trans. Pattern Anal. Mach. Intell..

[3] Hirose O. (2023). Geodesic-Based Bayesian Coherent Point Drift. IEEE Trans. Pattern Anal. Mach. Intell..

[4] Zhang Z., Dai Y., Sun J. (2020). Deep learning based point cloud registration: An overview. Virtual Real. Intell. Hardw..

[5] Min Z., Wang J., Pan J., Meng M.Q.H. (2021). Generalized 3-D Point Set Registration With Hybrid Mixture Models for Computer-Assisted Orthopedic Surgery: From Isotropic to Anisotropic Positional Error. IEEE Trans. Autom. Sci. Eng..

[6] De Silva V., Roche J., Kondoz A. (2018). Fusion of LiDAR and camera sensor data for environment sensing in driverless vehicles. arXiv.

[7] Giering M., Venugopalan V., Reddy K. Multi-modal sensor registration for vehicle perception via deep neural networks. Proceedings of the 2015 IEEE High Performance Extreme Computing Conference (HPEC).

[8] Mastin A., Kepner J., Fisher J. Automatic registration of LIDAR and optical images of urban scenes. Proceedings of the 2009 IEEE Conference on Computer Vision and Pattern Recognition.

[9] Rosas-Cervantes V., Lee S.G. (2020). 3D Localization of a Mobile Robot by Using Monte Carlo Algorithm and 2D Features of 3D Point Cloud. Int. J. Control. Autom. Syst..

[10] Lu W., Wan G., Zhou Y., Fu X., Yuan P., Song S. Deepvcp: An end-to-end deep neural network for point cloud registration. Proceedings of the IEEE International Conference on Computer Vision.

[11] Rundo L., Tangherloni A., Militello C., Gilardi M.C., Mauri G. Multimodal medical image registration using particle swarm optimization: A review. Proceedings of the 2016 IEEE Symposium Series on Computational Intelligence (SSCI).

[12] Chen Y., He F., Li H., Zhang D., Wu Y. (2020). A full migration BBO algorithm with enhanced population quality bounds for multimodal biomedical image registration. Appl. Soft Comput..

[13] Collignon A., Vandermeulen D., Suetens P., Marchal G. (1995). 3D multi-modality medical image registration using feature space clustering. Proceedings of the International Conference on Computer Vision, Virtual Reality, and Robotics in Medicine.

[14] Sinko M., Kamencay P., Hudec R., Benco M. 3D registration of the point cloud data using ICP algorithm in medical image analysis. Proceedings of the 2018 IEEE ELEKTRO.

[15] El-Hakim S.F., Beraldin J.A., Picard M., Godin G. (2004). Detailed 3D reconstruction of large-scale heritage sites with integrated techniques. IEEE Comput. Graph. Appl..

[16] Zhao X., Zhang C., Wang Y., Yang B. (2011). A hybrid approach based on MEP and CSP for contour registration. Appl. Soft Comput..

[17] Bermejo E., Cordón O., Damas S., Santamaría J. (2013). Quality time-of-flight range imaging for feature-based registration using bacterial foraging. Appl. Soft Comput..

[18] Ma J., Jiang J., Liu C., Li Y. (2017). Feature guided Gaussian mixture model with semi-supervised EM and local geometric constraint for retinal image registration. Inf. Sci..

[19] Keller M., Lefloch D., Lambers M., Izadi S., Weyrich T., Kolb A. Real-time 3D reconstruction in dynamic scenes using point-based fusion. Proceedings of the 2013 IEEE International Conference on 3D Vision-3DV.

[20] Zhang Z. (1994). Iterative point matching for registration of free-form curves and surfaces. Int. J. Comput. Vis..

[21] Conte D., Foggia P., Sansone C., Vento M. (2004). Thirty years of graph matching in pattern recognition. Int. J. Pattern Recognit. Artif. Intell..

[22] Gao W., Tedrake R. Filterreg: Robust and efficient probabilistic point-set registration using gaussian filter and twist parameterization. Proceedings of the IEEE Conference on Computer Vision and Pattern Recognition.

[23] Jian B., Vemuri B.C. (2010). Robust point set registration using gaussian mixture models. IEEE Trans. Pattern Anal. Mach. Intell..

[24] Tao W., Sun K. Asymmetrical Gauss mixture models for point sets matching. Proceedings of the IEEE Conference on Computer Vision and Pattern Recognition.

[25] Ravikumar N., Gooya A., Çimen S., Frangi A.F., Taylor Z.A. (2018). Group-wise similarity registration of point sets using Student’s t-mixture model for statistical shape models. Med Image Anal..

[26] Bouguila N., Fan W. (2020). Mixture Models and Applications.

[27] Sefidpour A., Bouguila N. (2012). Spatial color image segmentation based on finite non-Gaussian mixture models. Expert Syst. Appl..

[28] Hu C., Fan W., Du J., Bouguila N. (2019). A novel statistical approach for clustering positive data based on finite inverted Beta–Liouville mixture models. Neurocomputing.

[29] Bouguila N., Elguebaly T. (2012). A fully Bayesian model based on reversible jump MCMC and finite Beta mixtures for clustering. Expert Syst. Appl..

[30] Fan J., Yang J., Ai D., Xia L., Zhao Y., Gao X., Wang Y. (2016). Convex hull indexed Gaussian mixture model (CH-GMM) for 3D point set registration. Pattern Recognit..

[31] Pascal F., Bombrun L., Tourneret J.Y., Berthoumieu Y. (2013). Parameter estimation for multivariate generalized Gaussian distributions. IEEE Trans. Signal Process..

[32] Elguebaly T., Bouguila N. (2015). Model-based approach for high-dimensional non-Gaussian visual data clustering and feature weighting. Digit. Signal Process..

[33] Franchini S., Charogiannis A., Markides C.N., Blunt M.J., Krevor S. (2019). Calibration of astigmatic particle tracking velocimetry based on generalized Gaussian feature extraction. Adv. Water Resour..

[34] Kubo Y., Takamune N., Kitamura D., Saruwatari H. (2020). Blind Speech Extraction Based on Rank-Constrained Spatial Covariance Matrix Estimation With Multivariate Generalized Gaussian Distribution. IEEE/ACM Trans. Audio Speech Lang. Process..

[35] Hristova H., Le Meur O., Cozot R., Bouatouch K. (2017). Transformation of the multivariate generalized Gaussian distribution for image editing. IEEE Trans. Vis. Comput. Graph..

[36] Verdoolaege G., Scheunders P. (2011). Geodesics on the manifold of multivariate generalized Gaussian distributions with an application to multicomponent texture discrimination. Int. J. Comput. Vis..

[37] Rami H., Belmerhnia L., El Maliani A.D., El Hassouni M. (2016). Texture retrieval using mixtures of generalized Gaussian distribution and Cauchy–Schwarz divergence in wavelet domain. Signal Process. Image Commun..

[38] Verdoolaege G., Rosseel Y., Lambrechts M., Scheunders P. Wavelet-based colour texture retrieval using the Kullback–Leibler divergence between bivariate generalized Gaussian models. Proceedings of the 16th IEEE International Conference on Image Processing (ICIP).

[39] Channoufi I., Bourouis S., Bouguila N., Hamrouni K. (2018). Image and video denoising by combining unsupervised bounded generalized gaussian mixture modeling and spatial information. Multimed. Tools Appl..

[40] Channoufi I., Bourouis S., Bouguila N., Hamrouni K. Color image segmentation with bounded generalized gaussian mixture model and feature selection. Proceedings of the 4th IEEE International Conference on Advanced Technologies for Signal and Image Processing (ATSIP).

[41] Allili M.S., Bouguila N., Ziou D. Finite generalized Gaussian mixture modeling and applications to image and video foreground segmentation. Proceedings of the 4th IEEE Canadian Conference on Computer and Robot Vision (CRV’07).

[42] Najar F., Bourouis S., Bouguila N., Belghith S. (2019). Unsupervised learning of finite full covariance multivariate generalized Gaussian mixture models for human activity recognition. Multimed. Tools Appl..

[43] Najar F., Bourouis S., Bouguila N., Belghith S. A fixed-point estimation algorithm for learning the multivariate GGMM: Application to human action recognition. Proceedings of the 2018 IEEE Canadian Conference on Electrical & Computer Engineering (CCECE).

[44] Gebru I.D., Alameda-Pineda X., Forbes F., Horaud R. (2016). EM algorithms for weighted-data clustering with application to audio-visual scene analysis. IEEE Trans. Pattern Anal. Mach. Intell..

[45] Agarwal P., Jleli M., Samet B. (2018). Banach Contraction Principle and Applications. Fixed Point Theory in Metric Spaces.

[46] Figueiredo M.A.T., Jain A.K. (2002). Unsupervised learning of finite mixture models. IEEE Trans. Pattern Anal. Mach. Intell..

[47] Hershey J.R., Olsen P.A. Approximating the Kullback Leibler divergence between Gaussian mixture models. Proceedings of the 2007 IEEE International Conference on Acoustics, Speech and Signal Processing-ICASSP’07.

[48] Durrieu J.L., Thiran J.P., Kelly F. Lower and upper bounds for approximation of the Kullback–Leibler divergence between Gaussian mixture models. Proceedings of the 2012 IEEE International Conference on Acoustics, Speech and Signal Processing (ICASSP).

[49] Cui S., Datcu M. Comparison of Kullback–Leibler divergence approximation methods between Gaussian mixture models for satellite image retrieval. Proceedings of the 2015 IEEE International Geoscience and Remote Sensing Symposium (IGARSS).

